# Scorpion Venom Peptide Effects on Inhibiting Proliferation and Inducing Apoptosis in Canine Mammary Gland Tumor Cell Lines

**DOI:** 10.3390/ani11072119

**Published:** 2021-07-16

**Authors:** Kamonporn Panja, Supranee Buranapraditkun, Sittiruk Roytrakul, Attawit Kovitvadhi, Preeda Lertwatcharasarakul, Takayuki Nakagawa, Chunsumon Limmanont, Tassanee Jaroensong

**Affiliations:** 1 Department of Companion Animal Clinical Sciences, Faculty of Veterinary Medicine, Kasetsart University, Bangkhen Campus, Bangkok 10900, Thailand; Kamonporn.panj@ku.th (K.P.); L_chunsumon@hotmail.com (C.L.); 2 Faculty of Veterinary Medicine, Rajamangala University of Technology Tawan-ok, Bangpra, Chonburi 20110, Thailand; 3 Division of Allergy and Clinical Immunology, Department of Medicine, Faculty of Medicine, Chulalongkorn University, Bangkok 10330, Thailand; Supranee.B@chula.ac.th; 4 Center of Excellence in Vaccine Research and Development (Chula Vaccine Research Center-Chula VRC), Faculty of Medicine, Chulalongkorn University, Bangkok 10330, Thailand; 5 Thai Pediatric Gastroenterology, Hepatology and Immunology (TPGHAI) Research Unit, Faculty of Medicine, Chulalongkorn University, Bangkok 10330, Thailand; 6 Functional Ingredients and Food Innovation Research Group, National Center for Genetic Engineering and Biotechnology, National Science and Technology Development Agency, Pathum Thani 12120, Thailand; sittiruk@biotec.or.th; 7 Department of Physiology, Faculty of Veterinary Medicine, Kasetsart University, Bangkok 10900, Thailand; fvetawk@ku.ac.th; 8 Department of Pathology, Faculty of Veterinary Medicine, Kasetsart University, Kampaeng Saen Campus, Nakhon Pathom 73140, Thailand; preeda.le@ku.th; 9 Laboratory of Veterinary Surgery, Graduate School of Agricultural and Life Sciences, The University of Tokyo, 1-1-1 Yayoi, Bunkyo-ku, Tokyo 113-8657, Japan; anakaga@g.ecc.u-tokyo.ac.jp

**Keywords:** apoptosis, BmKn-2, canine mammary gland tumor, scorpion venom peptide

## Abstract

**Simple Summary:**

Canine mammary gland tumors (CMGTs) are quite common in intact female dogs. In diagnosed cases, approximately 50% of mammary gland tumors metastasized. Chemotherapy is a practical treatment to increase the median survival time, but it has severe side effects and impacts on resistance. BmKn-2, an antimicrobial peptide derived from scorpion venom, has displayed anticancer effects in oral and colon human cancer cell lines. Consequently, BmKn-2 may be useful in the molecular treatment of CMGTs. This study investigated the effect of BmKn-2 on the proliferation inhibition, apoptotic induction and the related mechanisms of those actions in CMGT cell lines, metastatic (CHMp-5b) and non-metastatic (CHMp-13a). The experimental results showed that BmKn-2 effectively inhibited proliferation and induced apoptosis in both CMGT cell lines via Bcl-2 (B-cell lymphoma-2) down-regulation and Bax (Bcl2 associated X) up-regulation gene expressions. Therefore, BmKn-2 could be used as a candidate molecular treatment for CMGTs in the future.

**Abstract:**

The most common neoplasms in intact female dogs are CMGTs. BmKn-2, an antimicrobial peptide, is derived from scorpion venom and has published anticancer effects in oral and colon human cancer cell lines. Thus, it is highly likely that BmKn-2 could inhibit CMGT cell lines which has not been previously reported. This study investigated the proliferation and apoptotic properties of BmKn-2 via Bax and Bcl-2 relative gene expression in two CMGT cell lines, metastatic (CHMp-5b) and non-metastatic (CHMp-13a). The results showed that BmKn-2 inhibited proliferation and induced apoptosis in the CMGT cell lines. The cell morphology clearly changed and increased apoptosis in a dose dependent of manner. The half maximum inhibitory concentration (IC_50_) was 30 µg/mL for CHMp-5b cell line and 54 µg/mL for CHMp-13a cell line. The induction of apoptosis was mediated through Bcl-2 and Bax expression after BmKn-2 treatment. In conclusion, BmKn-2 inhibited proliferation and induced apoptosis in both CHMp-5b and CHMp-13a cell lines via down-regulation of Bcl-2 and up-regulation of Bax relative mRNA expression. Therefore, BmKn-2 could be feasible as candidate treatment for CMGTs.

## 1. Introduction

The most common group of neoplasms in intact female dogs is canine mammary gland tumors (CMGTs) [1,2]. The majority malignancy of mammary gland tumors is encountered in reproductively intact female dogs [3]. Approximate 50% of mammary gland tumors metastasize when the dogs are diagnosed [4]. Surgery is the current treatment of choice of CMGTs. However, the malignant tumors with distant metastasis or ineffectively treated with local therapy alone may be palliated with chemotherapy, such as 5-fluorouracil (5-FU) and cyclophosphamide. The 5-FU affects to inhibit cell growth and cell division leads to cell death especially at S-phase of cell cycle. Cyclophosphamide, alkylating agent, induces cytotoxic effect and interfering with DNA replication and RNA transcription [5,6]. Even though chemotherapy is a practical treatment to increases the median survival time [7], it has severe side effects and impacts on resistance. In Chinese traditional medicine, scorpion venom has been used to treat some neurological diseases such as stroke, tetanus and rheumatism [8] and it can reduce many symptoms, for example, seizure, cardiovascular diseases, pain and tumors for sustained periods [9]. Many peptides in scorpion venom have been identified and their antimicrobial activities have been reported [10]. Antimicrobial peptides (AMPs), as host defense peptides, can diminish infections in animals and humans. Other benefits of AMPs are as vectors of drug transport and anticancer properties [11,12]. BmKn-2 is an antimicrobial peptide which is extracted from the venom of the scorpion *Mesobuthus martensii Karsch* [13]. Previous experiments using BmKn-2 showed that BmKn-2 induced apoptosis in oral cancer cells in humans [14,15]. BmKn-2 inhibited the growth of human colon cancer cells and did not induce red blood cell hemolysis [16,17]. Therefore, BmKn-2 has high potential to inhibit CMGTs; however, this use has not yet been reported and the apoptosis mechanism induced by BmKn-2 in CMGTs is still unknown. The properties of inhibited proliferation and apoptosis can interrupt the hall mark of cancer [18]. In the apoptosis pathway, the Bcl-2 gene family mostly regulates this pathway [19] and controls mitochondrial integrity in cells [20]. The Bcl-2 gene family can be divided into 2 parts based on their actions: (1) activating apoptosis processes such as Bax, Bad, Bak and Bok; and (2) stopping apoptosis processes such as Bcl-2, Bcl-XL and Bcl-W [21]. Especially, the expression of Bax and Bcl-2 mRNA are generally approved as apoptosis indicators [22]. Thus, the study of these properties and mechanism in BmKn-2 and their effect on CMGTs is very important for developing new antitumor substances in the future. In addition, CMGTs model can be a valuable method for the evaluation and development of novel medicines and their therapeutic strategies and would be advantageous for dogs and human cancer patients in the future [23,24]. The aim of this study was to inspect the proliferation and apoptotic properties of BmKn-2 via Bax and Bcl-2 relative gene expression in CMGT cell lines.

## 2. Materials and Methods

### 2.1. Antimicrobial Peptides (AMPs)

BmKn-2 peptide was derived from China Peptides Corporation (LTD; Shanghai, China). The molecular weight of the peptide was 1448.81 Daltons, and its polymer unit was 13. The net charge of BmKn-2 was +2 and its hydrophobicity was 56.23% [13]. BmKn-2 was dissolved in dimethyl sulfoxide (DMSO; Sigma-Aldrich, Singapore) and the solution was diluted in culture medium RPMI 1640 (Corning, NY, USA) to obtain the required concentrations.

### 2.2. Cell Culture

The CMGT cell lines were obtained from the Laboratory of Veterinary Surgery, Graduate School of Agricultural and Life Sciences, The University of Tokyo. Two canine mammary gland tumor cell lines; CHMp-5b (metastatic cell line) and CHMp-13a (non-metastatic cell line) were chosen to use in this research [25]. Both cell lines were cultured in RPMI 1640 medium supplemented with 10% heat-inactivated fetal bovine serum (Gibco; Waltham, MA, USA) and 1% penicillin/streptomycin (Gibco; Waltham, MA, USA). These cell lines were incubated at 37 °C with 5% CO_2_ before use [15].

### 2.3. Cell Viability Assay

MTT assay was used to assess cell viability. The CHMp-5b and CHMp-13a cell lines were cultured overnight in 96 well-plates with 5000 cells per well. Treatments were in triplicate with various doses (0, 6.25, 12.5, 25, 50, 100 µg/mL) of BmKn-2, and they were incubated for 24 h at 37 °C with 5% CO_2_. After that, MTT (3-[4,5-dimethylthiazol-2-yl]-2,5-diphenyl tetrazolium bromide) labeling reagent 10 µL (Roche Diagnostics GmbH; Mannheim, Germany) was added into each well. The cell lines were incubated in microplates for 4 h at 37 °C with 5% CO_2_ in humidified incubator. Next, the cell lines were added 100 µL the solubilization solution (10% sodium dodecyl sulfate in 0.01 M HCl) into each well. A solvent control was made of cells treated with 0.1% DMSO, while the optical density (OD) control was untreated cells. The microplates were incubated overnight before OD measurement at 580 nm using a microplate reader.

### 2.4. Annexin V/Propidium Iodide (PI) Apoptosis Analysis by Flow Cytometry

The CHMp-5b and CHMp-13a cell lines were cultured overnight in 6-well plates at 200,000 cells per well and were treated with BmKn-2 at 0, 15, 30, 60 µg/mL in triplicate for 24 h. The cell lines were stained using FITC-labeled-Annexin V and PI (Biolegend; San Diego, CA, USA) and incubated for 15 min at room temperature in the dark, according to the manufacturer’s protocols. The number of apoptotic cells was evaluated using flow cytometry (BD FACS Calibur; BD Biosciences; San Jose, CA, USA). The cells were collected and evaluated using the CellQuestPro software (version 5.0; BD Biosciences; San Jose, CA, USA) and apoptotic cells were indicated using a FACS Calibur with the CellQuest Pro Software (BD Biosciences; San Jose, CA, USA). The experiments were carried out in triplicate.

### 2.5. RNA Isolation and Real-Time PCR

The total RNA extraction was carried out using TRIZOL reagent (Invitrogen; Carlsbad, CA, USA) for all cell lines, treated with BmKn-2 peptide at 0, 15, 30, 60 µg/mL. The extracted RNA concentration of each treatment was 1 µg/µL. The cDNA was synthesized from purified RNA using iScript Reverse Transciption Supermixed for Real-time PCR (Bio-Rad; Foster, CA, USA). Real-time PCR was performed using iTaq Universal SYBR Green Supermix (Bio-Rad; Foster, CA, USA) on Bio-Rad CFX Connect Real-Time system. Primer sequences used for real-time PCR were Bax, Bcl-2, RPS-19 (ribosomal protein S19) and GAPDH (glyceraldehyde 3-phosphate dehydrogenase) [26] (Table 1). The gene expression was analyzed using the Biorad CFX manager^TM^ software (Bio-Rad; Hercules, CA, USA). The reference genes, GAPDH and RPS-19, were used to normalize the expression of the apoptotic genes. The relative mRNA expression level was calculated using the 2^−ΔΔCT^ method [27] and the experiments were performed in triplicate.

### 2.6. Statistical Analysis

The experiments were performed in triplicate and the data presented as mean ± standard deviation. The significance of differences was evaluated using one-way ANOVA followed by Scheffé’s multiple comparison test as post-hoc analysis for which groups were fixed factors using the R-statistic program (R Development Core Team, 2008) and under R Studio ver. 1.4.1103 with the Rcmdr package. The normal distribution and homogeneity of variance were confirmed using the Shapiro-Wilk test and Levene’s test, respectively. *p*-values less than 0.05 were considered significantly different.

## 3. Results

### 3.1. Effect of BmKn-2 on Cell Viability of CMGT Cell Lines

#### 3.1.1. Effect of BmKn-2 on Percentage Viability of CHMP-5b and CHMP-13a Cell Lines

**Figure 1:**
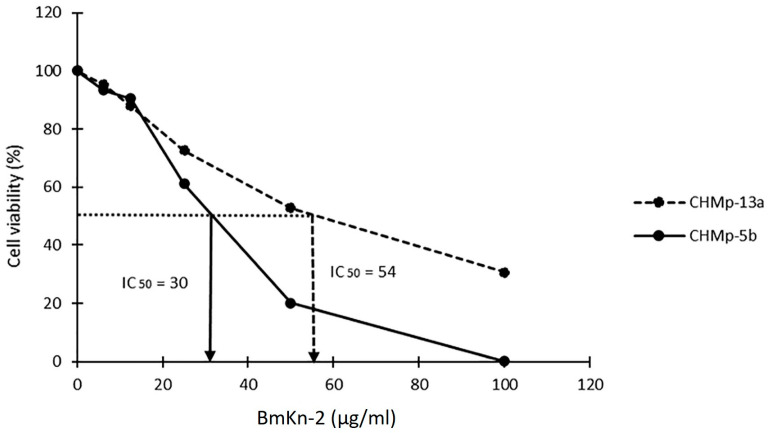
Percentage cell viability in CHMp-5b and CHMp-13a cell lines, treated with BmKn-2 (0–100 µg/mL) for 24 h.

The CHMp-5b and CHMp-13a cell lines were treated with different concentrations of BmKn-2 (0–100 µg/mL) for 24 h. Cell viability was determined based on MTT assay. The half maximum inhibitory concentration (IC_50_) values at 24 h of CHMp-5b and CHMp-13a were 30 ± 1.32 µg/mL and 54 ± 2.03 µg/mL, respectively (Figure 1).

#### 3.1.2. Effect of BmKn-2 on Viability and Morphology of CHMp-5b and CHMp-13a Cell Lines

**Figure 2:**
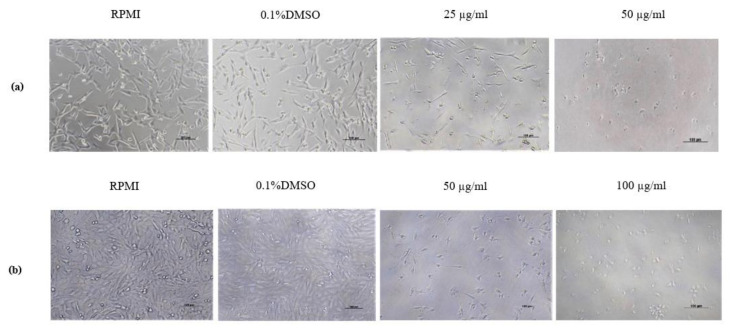
Cell morphology of cell lines in RPMI, 0.1% DMSO, BmKn-2 at 25, 50 and 100 µg/mL for 24 h: (**a**) CHMp-5b; (**b**) CHMp-13a. Cells illustrated using phase-contrast microscopy at 100× magnification; scale bar = 100 µm.

Applying BmKn-2 at 25 and 50 µg/mL in CHMp-5b cell line resulted in cell shrinkage, rounding, loss of cell-to-cell adhesion and some cells were detached from the tissue culture plate (Figure 2a), while cell morphologies in RPMI and 0.1% DMSO remained normal shapes. With CHMp-13a, the cell morphology changed by adding BmKn-2 at 50 µg/mL and 100 µg/mL (Figure 2b).

### 3.2. Effect of BmKn-2 on Apoptosis Induction in CMGT Cell Lines

**Figure 3:**
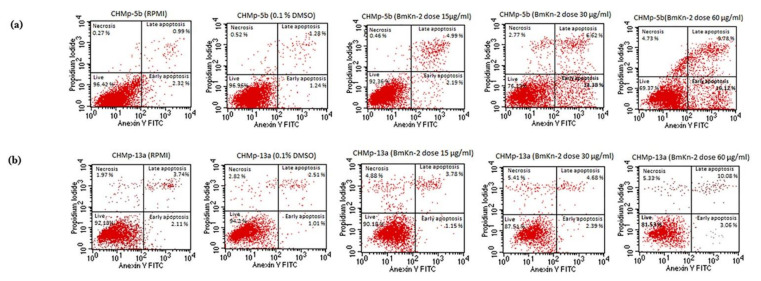

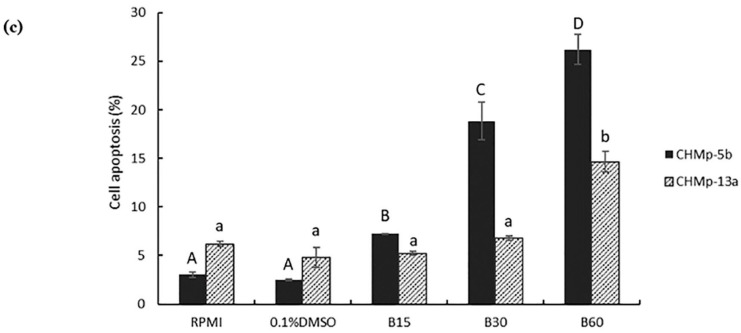
Flow cytometry image of CMGTs in RPMI, 0.1% DMSO and BmKn-2 at 15, 30, 60 µg/mL at 24 h: (**a**) CHMp-5b; (**b**) CHMp-13a; (**c**) Apoptosis percentages of BmKn-2 at 15, 30 and 60 µg/mL significantly increased apoptosis percentage in CHMp-5b, the apoptosis percentage of CHMp-13a significantly increased by treating BmKn-2 at 60 µg/mL where different letters on superscripts between plots representing the statistically significant differences (*p* < 0.05).

The percentage apoptosis was obtained using flow cytometry. The CHMp-5b and CHMp-13a cell lines were treated with BmKn-2 at 0, 15, 30 and 60 µg/mL for 24 h. Cell death stages were determined by the different labelling patterns in the annexin V/PI stain. Live cells were identified by both Annexin V- and PI-negative stain cells. Early apoptotic cells were categorized based on Annexin V-positive and PI-negative stain cells. Cells in late apoptosis were classified by both annexin V and PI-positive stain cells. Necrotic cells were indicated by annexin V-negative and PI-positive stain cells (Figure 3a,b). The apoptosis percentage of CHMp-5b increased from 2.49% in 0.1% DMSO (control group) to 7.23%, 18.84% and 26.21% by adding BmKn-2 at 15, 30 and 60 µg/mL, respectively (Figure 3c). Applying BmKn-2 at 15, 30 and 60 µg/mL significantly increased apoptosis percentage in CHMp-5b. For CHMp-13a, the apoptosis percentage increased from 4.78% in 0.1% DMSO (control group) to 14.65% by adding BmKn-2 at 60 µg/mL (Figure 3c). The apoptosis percentage of CHMp-13a significantly increased by treating BmKn-2 at 60 µg/mL. On the other hand, using BmKn-2 at 15 and 30 µg/mL did not affect the apoptosis percentage in CHMp-13a. In addition, the apoptosis percentage of cell lines in RPMI was not different compared to 0.1% DMSO, suggesting that 0.1% DMSO did not affect the apoptosis level of those cell lines.

### 3.3. Effect of BmKn-2 on Apoptosis Induction and Identify Apoptosis Pathways in CMGT Cell Lines

**Figure 4:**
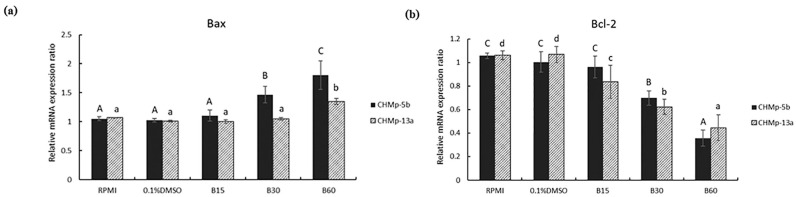

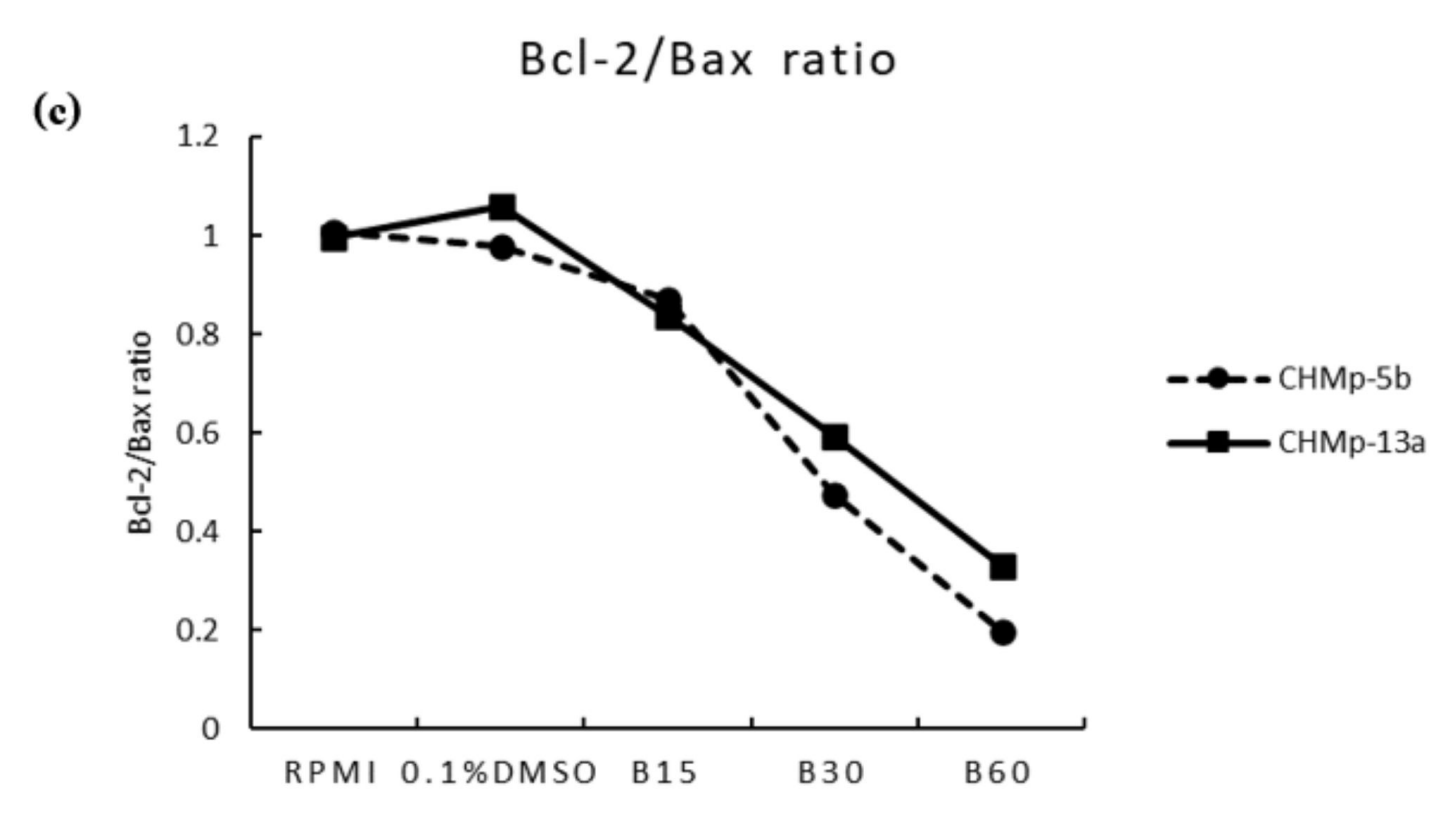
mRNA expression ratios of CHMp-5b and CHMp-13a cell lines in RPMI, 0.1% DMSO, BmKn-2 at 15, 30 and 60 µg/mL: (**a**) The Bax mRNA expression ratios of CHMp-5b treated by adding BmKn-2 at 30 and 60 µg/mL were significantly higher than control group, while the Bax mRNA expression ratios of CHMp-13a with BmKn-2 added at 60 µg/mL was significantly different from the control; (**b**) The Bcl-2 mRNA expression ratios of CHMp-5b added with BmKn-2 at 30 and 60 µg/mL were clearly significantly lower than control; CHMp-13a, the relative Bcl-2 mRNA expression ratios decreased significantly with BmKn-2 at 15, 30 and 60 µg/mL; (**c**) Ratio of Bcl-2 to Bax where different letters on superscripts between plots representing the statistically significant differences (*p* < 0.05).

The relative mRNA expression ratios of Bax and Bcl-2 in CHMp-5b and CHMp-13a cell lines were observed using RT-PCR after those cell lines had been treated with BmKn-2 at 0, 15, 30 and 60 µg/mL for 24 h. The results showed that relative mRNA expression ratios of Bax in CHMp-5b increased from 1.03 in 0.1% DMSO (control group) in to 1.47 and 1.80 by adding BmKn-2 at 30 and 60 µg/mL (Figure 4a). With CHMp-13a, the relative mRNA expression ratios of Bax increased from 1.01 in 0.1% DMSO (control group) to 1.35 after treating by adding BmKn-2 at 60 µg/mL. The Bax mRNA expression ratios of CHMp-5b treated by adding BmKn-2 at 30 and 60 µg/mL were significantly higher than control group, while the Bax mRNA expression ratios of CHMp-13a with BmKn-2 added at 60 µg/mL was significantly different from the control (Figure 4a). The relative Bcl-2 mRNA expression ratios of CHMp-5b decreased from 1.00 in 0.1% DMSO (control group) to 0.7 and 0.36 by adding BmKn-2 at 30 and 60 µg/mL (Figure 4b). With CHMp-13a, the relative Bcl-2 mRNA expression ratios decreased from 1.07 in 0.1% DMSO (control group) to 0.84, 0.62 and 0.45 after treating with BmKn-2 at 15, 30 and 60 µg/mL, respectively. The Bcl-2 mRNA expression ratios of CHMp-5b added with BmKn-2 at 30 and 60 µg/mL were clearly significantly lower than control (Figure 4b). Both the Bax and Bcl-2 mRNA expression ratios in RPMI were similar to those for 0.1% DMSO in the cell lines. The ratios of Bcl-2 to Bax mRNA expressions in the cell lines were reduced with increasing BmKn-2 doses (Figure 4c).

## 4. Discussion

In this study, the MTT assay results demonstrated the cytotoxicity of BmKn-2 in CMGT cell lines, CHMp-5b and CHMp-13a. The IC_50_ of BmKn-2 in CHMp-5b was 30 µg/mL which was nearly the IC _50_ of this peptide in human oral squamous carcinoma (HSC4) and mouth epidermoid carcinoma (KB) cells were 26 µg/mL and 34 µg/mL [15]. Furthermore, the IC_50_ of CHMp-13a was 54 µg/mL which was close to the value reported in a study of the peptide on colon cancer cell lines (SW620) was 40 µM [16]. BmKn-2 could attach the surface of cancer cells because of a massive number of lactate anions were secreted from the cancer cells and crossed the plasma membranes as a hallmark. This mechanism tended to generate the unique negative charges on the surfaces of them [28] while BmKn-2 had positive net charge [13]. Therefore, the opposite of charges attracts them together. This reason supported that the potential BmKn-2 action inhibited cancer cell proliferation and induced apoptosis via Bcl-2 down-regulation and Bax up-regulation gene expressions.

Apoptosis is an effective mechanism to control malignant cells, inhibit hyperplasia, stop tumor progression and terminate carcinogenesis [29,30]. Manipulating the apoptosis induction pathway is the key in cancer therapy [31]. Moreover, apoptotic cell death is not to create an inflammatory reaction to nearby cells because phagocytes come and wrap apoptotic cells to protect the cellular components from leaking out [32,33]. In this study, BmKn-2 at 30 µg/mL was used to treat in CMGT cell lines, it induced many apoptosis cells and found a few necrotic cells which were similar to the report Satitmanwiwat et al., 2014 [15,16]. These results suggested that BmKn-2 induced apoptosis cell death and created less inflammatory response. On the other hand, late apoptotic and necrotic cell death created pro-inflammatory danger signal emission, but early apoptotic cell death made a specific signal to call phagocytes without causing inflammation [34,35]. The CHMp-5b cells (BmKn-2 at dose 30 µg/mL) found that the percentage of early apoptosis was more than late apoptosis and necrosis. In contrast, the percentage of early apoptosis in CHMp-13a cells was less than late apoptosis and necrosis. Thus, using BmKn-2 on the CHMp-5b cell lines released less pro-inflammatory danger signals than for the CHMp-13a cell lines. This information suggested that BmKn-2 might be appropriate as the candidate treatment for CMGTs.

This research also studied BmKn-2 apoptosis-induction mechanisms in CMGT cell lines by observing the expression of Bax and Bcl-2 mRNA, which are generally approved apoptosis indicators [22]. The Bcl-2 gene family mostly regulates the apoptosis pathway [19] and controls mitochondrial integrity in cells [20]. The Bcl-2 gene family can be divided into 2 parts based on their actions: (1) activating apoptosis processes such as Bax, Bad, Bak and Bok; and (2) stopping apoptosis processes such as Bcl-2, Bcl-XL and Bcl-W [21]. Other studies have reported that anti-apoptosis action occurs when the Bcl-2 gene is overexpressed the formation of Bcl-2/Bax heterodimers in apoptotic cell death [22,32]. The properties of Bcl-2 genetic in dog may help to find the drug designing in cancers [36]. In addition, it could be used as potential goal on CMGT therapeutics in the future. Thus, Bcl-2 gene expression is a worthy target for CMGT treatments [37]. The Bcl-2 overexpression stops configuration changes and the translocation of Bax expression [38,39]. In the current study, the real-time PCR results showed that BmKn-2 increased Bax mRNA expression but reduced Bcl-2 mRNA expression. This supported the other research that showed BmKn-2 induced apoptosis in human oral cancer cells and colon cancer cells through Bax up-regulation and Bcl-2 down-regulation expressions [14,15]. Furthermore, BmKn-2 decreased the ratio of Bcl-2 to Bax mRNA expression. This ratio is a vital indicator that cells are more susceptible to apoptosis [40]. The lower the Bcl-2 to Bax ratio mRNA expression, the more apoptotic inductions occurred [41].

## 5. Conclusions

The experimental results demonstrated that BmKn-2 effectively inhibited proliferation and induced apoptosis in CMGT cell lines, metastatic (CHMp-5b) and non-metastatic (CHMp-13a) via the Bax up-regulation and Bcl-2 down-regulation mRNA expression pathways. BmKn-2 increased more apoptosis in metastatic CHMp-5b CMGT cells when compared to non-metastatic CHMp-13a CMGT cells. However, there were few necrotic cells in both CMGT cell lines. These results suggested that BmKn-2 induced apoptotic cell death with less inflammatory response. Therefore, BmKn-2 has potential for use as a novel candidate treatment for CMGTs. Further study should investigate in vivo experiments using BmKn-2 and the development of practical treatments.

## Figures and Tables

**Table 1:** Primer sequences for real-time PCR.

Gene	Primer Sequence (5′to3′)	Annealing	Product Size (bp ^1^)	Accession Number
*Bcl-2*	Forward: TGGATGACTGAGTACCTGAA Reverse: GGCCTACTGACTTCACTTAT	59	206	AB116145
*Bax*	Forward: GGTTGTTGCCCTCCTCTACT Reverse: GTAAGCACTCCAGCCACAAA	59	219	AB080230
*GAPDH*	Forward: TGTCCCCACCCCCAATGTATC Reverse: CTCCGATGCCTGCTTCACTACCTT	59	100	NM 001003142
*RPS-19*	Forward: CCTTCCTCAAAAAGTCTGGG Reverse: GTTCTCATCGTAGGGAGCAAG	59	95	XM 533657.3

^1^ base pair.

## Data Availability

The data presented in this study are available within the article. Raw data supporting this study are available from the corresponding author.
